# Implementation of a Mental Health Care Package for Children in Areas of Armed Conflict: A Case Study from Burundi, Indonesia, Nepal, Sri Lanka, and Sudan

**DOI:** 10.1371/journal.pmed.1001371

**Published:** 2013-01-15

**Authors:** Mark J. D. Jordans, Wietse A. Tol, Dessy Susanty, Prudence Ntamatumba, Nagendra P. Luitel, Ivan H. Komproe, Joop T. V. M. de Jong

**Affiliations:** 1 Research and Development Department, HealthNet TPO, Amsterdam, the Netherlands; 2 Centre for Global Mental Health, London School of Hygiene and Tropical Medicine, London, United Kingdom; 3 Department of Mental Health, Johns Hopkins Bloomberg School of Public Health, Baltimore, Maryland, United States of America; 4 HealthNet TPO South Sudan, Wau, South Sudan; 5 APRODEM-Girizina, Bujumbura, Burundi; 6 Transcultural Psychosocial Organization Nepal, Kathmandu, Nepal; 7 Faculty of Social and Behavioural Sciences, Utrecht University, Utrecht, the Netherlands; 8 VU University Medical Center, Vrije Universiteit, Amsterdam, the Netherlands; 9 School of Medicine, Boston University, Boston, Massachusetts, United States of America

## Abstract

As one article in an ongoing series on Global Mental Health Practice, Mark Jordans and colleagues describe their work developing and evaluating a community-based psychosocial and mental health care package for children in five conflict affected countries: Burundi, Indonesia, Nepal, Sri Lanka, and Sudan.

**Summary Points**

- In the absence of existing mental health services, a newly developed community-based psychosocial and mental health care package for children was implemented and evaluated in five conflict-affected countries: Burundi, Indonesia, Nepal, Sri Lanka, and Sudan.
- Routine monitoring and evaluation combined with rigorous research design allowed for improvement and fine-tuning of services in real-life settings, and highlighted key gaps in current knowledge.
- The program has resulted in improved case detection with a developed and validated screening instrument, making care accessible to over 96,000 children, and generating empirical evidence on the effectiveness of interventions.
- Future development requires broadening the scope of the care package (i.e., integration of treatment for severe mental disorders, stronger involvement of families, and strengthening of primary prevention approaches) and continued evaluation of new elements.

*This case study is part of the* PLOS Medicine *series on Global Mental Health Practice.*

## Background

A recent review demonstrated that mental health problems are a leading cause of health-related disability in children and adolescents worldwide [1]. Even though children and adolescents constitute around 50% of the population in low- and middle-income countries (LMICs), their mental health needs are generally neglected [2],[3]. This is especially the case in settings where children are surrounded by perpetual violence and poverty [4],[5]. Violence may impact mental health and broader aspects of psychological and social well-being [4],[6]. This situation requires an intervention response that is comprehensive, as well as affordable, effective, and feasible to scale up.

Recent reviews have demonstrated that the evidence base for effective mental health interventions for children in LMICs, and specifically for children in areas of armed conflict, is slowly accruing [3],[7],[8]. Moreover, international guidelines have been developed to support establishment of services in LMICs [9],[10]. One core question is how to translate existing knowledge and guidelines into effective practice, and how to translate real-life practice into replicable and sustainable models that can be scaled up in other settings. This question is further complicated in conflict-affected areas and fragile states, because of severely disrupted health and community care structures [11] and the scarcity of effective and feasible service delivery models [7]. These challenges are emphasized by a recent review reporting a large gap between research and practice in humanitarian settings [8]. Globally, research–practice mismatches contribute to the limited availability and impact of child mental health interventions [2],[12]. McLennan and colleagues distinguish four types of research–practice gaps: “(1) the failure to implement interventions found to be effective, and the implementation of interventions (2) that have been demonstrated to cause harm, (3) that have evidence of no effect, and (4) where the effectiveness is unknown because of the lack of rigorous evaluation” [12].

The present case study describes an effort to address these challenges through an integrated intervention and research program. At the start of the program we were faced with a vast disparity between the number of children with psychosocial and mental health problems and the availability of evidence-based interventions in settings of armed conflict. Consequently, we followed a combined research–practice approach that aimed to overcome that disparity. We present a summary of the multi-tiered psychosocial and mental health care package, implemented between 2004 and 2010 in five countries [13]. We synthesize the interplay between research and practice in this program according to a set of principles extracted from guidelines on psychosocial support for children in areas of conflict [5],[14],[15] and emergency settings [9]. We conclude with recommendations to improve future uptake of psychosocial and mental health care. While several articles have been published on separate studies of the program [13],[16]–[21], we have thus far not provided an integrated overview of the overarching lessons learned through research and practice reflections.

## Case Study of a Care Package

### Development

The Child Thematic Program started in September 2004 in Burundi, Sudan, Sri Lanka, and Indonesia. Nepal was added to the program in July 2006. From the start, the care package was based on a public health model to include prevention, treatment, and rehabilitation interventions. The program was implemented through a number of partner organizations (Church World Services, Indonesia; Shantiham, Sri Lanka; Transcultural Psychosocial Organization, Nepal; HealthNet TPO, Burundi and Sudan).

A group of facilitators received a brief training (15–20 days) for the community-level activities and Classroom-Based Intervention (CBI). Locally available counselors were trained (approximately three months) to deliver mental health interventions to children in need of further indicated care. See Table 1 for information on the context and program in each country.

**Table 1:** Program implementation details for each country.

Characteristic	Country				
	Burundi	Sri Lanka	South Sudan	Indonesia	Nepal
Program sites	Three northwestern provinces where hostilities continued until 2009	Three educational zones in and around Jaffna (northern Sri Lanka)	Three *Payams* in Yei County, South Sudan	Five sub-districts of Poso, Central Sulawesi Province	Fourteen far- and mid-western districts of Nepal; 528 schools
Conflict	Burundi has been affected by killings and violence along ethnic and regional lines, re-erupting in a civil war from 1993 until 2003. Fighting between the Tutsi-dominated national army and rebel groups from the Hutu majority killed 300,000 and displaced over 1 million people.	In 1983, the LTTE launched an armed struggle for a Tamil homeland, because of perceived discrimination by the Sinhalese government; the conflict effectively ended when the Sri Lankan military defeated the LTTE in May 2009.	The civil war between the Islamist central government and peripheral areas represented by the Sudan People's Liberation Movement/Army formally ended in the Comprehensive Peace Agreement in 2005.	Recurring violence since 1998 as a result of hostility between Muslim and Protestant populations (caused by changed economic relations, migration, and state restructuring). Major hostilities lasted until 2001, with unrest and incidences of violence continuing up to 2007.	In 1996, the Communist Party of Nepal (Maoists) announced a “people's war” against the government of Nepal, which ended in November 2006 with a comprehensive peace agreement.
Beneficiaries	35,266	6,914	19,164	10,410	24,964
Cost per user (€)	5.31	6.66	13.67	16.37	NA

LTTE, Liberation Tigers of Tamil Eelam; NA, not available.

### Implementation

**Figure 1:**
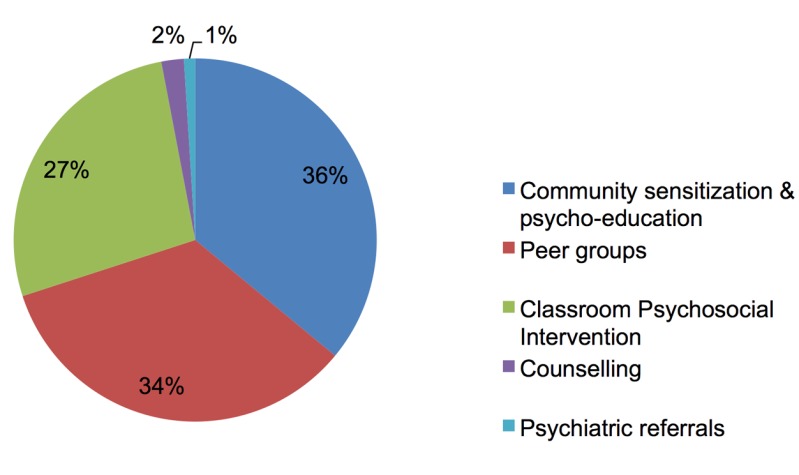
Service uptake of core interventions within the care package.

The multi-tiered care package consisted of a variety of interventions. The first tier comprised mental health promotion activities, aiming to increase adaptive adjustment and community resilience. This tier's activities included running peer groups, which were recreational activities combined with theme-centered group discussions for children without indication for care. Also, it included community sensitization and psycho-education to increase awareness of the mental health needs of children, as well as of existing coping strategies and resources. The second tier consisted of interventions that targeted subgroups of children with elevated psychosocial distress. This tier included a structured group intervention (CBI) aimed at decreasing symptoms of distress and strengthening protective factors. The third tier comprised treatment for children with severe mental health problems. This tier's actions included providing individual counseling to reduce symptoms and improve functioning. Children requiring specialized treatment were referred to a psychiatrist by the counselors, whenever necessary and possible. These interventions are further explained below, and Figure 1 presents the distribution of uptake of these interventions within the program (all sites combined).

The first step in implementing the care package was to introduce the program to the communities and assess needs through qualitative research [11]. This stage involved receiving approvals from local stakeholders, conducting community awareness and sensitization sessions about the psychosocial and mental health needs of children, enlisting community participation and ownership, and increasing empowerment in dealing with the problems of youth.

In the second step, all children in schools in the program's catchment areas were screened for psychosocial distress using a newly developed instrument (see below) [16],[22]. A prescreening briefing for children and parents was conducted to provide information on the program's objective and process (i.e., availability of different interventions). Furthermore, this briefing aimed to reduce the risk of stigma being associated with the screening.

In the third step, children scoring above a screening cutoff were offered CBI. This standardized intervention consisted of 15 sessions of 1.5 hours over five weeks involving cognitive behavioral techniques (psycho-education, strengthening coping, and guided exposure to past traumatic events through drawing) and creative expressive elements (cooperative games, structured movement, music, drama, and dance) with groups of around 15 children. All other children were offered the opportunity to participate in structured recreational peer group programs (these included sports activities, meditation and yoga, and games), including a psycho-educational element, taking place in the schoolyard. These groups served as an alternative intervention to CBI, to reduce stigma and to strengthen existing resilience by encouraging social support systems and normalization.

In the fourth step, children with sustained and serious mental health problems after termination of CBI were referred to the next tier of the system: problem-solving counseling, either individually or with their families (parental psycho-education sessions). The average number of counseling sessions ranged from 2.2 to 7.5, depending on the country. Throughout the program, biweekly supervision by a psychologist or senior counselor was provided for both the facilitators of CBI and the counselors providing treatment for children with more severe problems.

In the fifth step, children identified by counselors as having psychiatric problems were referred to a mental health specialist (if available), with follow-up and case management by the counselor. To maximize access to care, most interventions were offered within a school setting and implemented by people trained in the communities in which the interventions were implemented. Offering services outside the health sector was intended to minimize levels of stigma, and to increase involvement and mutual collaboration in often fragmented local communities. In this way, access to care was available to 96,718 children and parents.

## Impact and Barriers: The Interplay between Practice and Research

Beyond the implementation of services per se, the program aimed to work towards an evidence-based approach to the provision of psychosocial and mental health services. This was achieved by combining practice-based evidence, generated through an elaborate monitoring and evaluation system, with a number of scientific studies embedded within real-life practice. The interaction between research and practice allowed for continuous fine-tuning or adaptation of the care model being implemented. Table 2 summarizes the mutual influence of research and implementation, based on four identified key principles: multileveled care, effectiveness, context sensitivity, and resilience. Table 2 also provides an overview of the main outputs stemming from the program.

**Table 2:** Presentation of practice and research.

Key Principles	Program and Research Components	Lessons Learned from Interplay between Practice and Research
Multi-layered support system	Service provision to 96,718 children and parents, including provision of counseling, peer support groups, a group-based psychosocial intervention, and psycho-education.	Implementing a multi-tiered system of care is feasible. The program resulted in high levels of satisfaction among recipients and providers. Still, therapist burden is a serious concern, and while mean cost per service user (average US$8.40) is relatively low, it currently exceeds available government budgetary mental health allocation. The cost-per-user analyses led to country-specific recommendations for reducing cost.
	Practice-driven evaluation among recipients of services (*n = *29,292) assessing access to care, treatment satisfaction, therapist burden, and cost [16].	To increase access and inclusion, the program provided services outside the health sector, allowing for non-stigmatized and easy-access care, combined with screening as a strategy for case detection.
	Development and validation of a brief multidimensional instrument (Child Psychosocial Distress Screener) in Burundi (*n = *2,240), and cross-cultural construct validation in four countries (*n = *10,019) [17],[22].	
Effectiveness	Cluster randomized controlled trials comparing the 15-session CBI in Indonesia (*n = *495), Sri Lanka (*n = *399), and Nepal (*n = *325) [18],.	There is overall support for a task-shifting model, but effectiveness of treatment is not confirmed everywhere or for everyone.
	Controlled pre- and post-participation evaluation of parental psycho-education in reducing emotional and behavioral problems among school-aged children in Burundi (*n = *120) [29].	When existing care is ineffective or new interventions are required, a strategy that encompasses global and local knowledge on needs, feasibility, and effectiveness can be employed to develop components-based treatments [23].
	Series of 17 empirically grounded single case studies in Burundi and Sudan to assess whether treatment is associated with client changes and to generate hypotheses on key treatment processes of counseling [21].	
Context sensitivity	Qualitative research to study the impact of communal violence on the psychosocial well-being of children in Indonesia [11].	Systematic attention to adaptation of interventions within different cultural contexts is required to increase acceptability within host communities.
	Development of context-specific instruments, for example, to assessment impairment in daily functioning [30].	Psychosocial programs should focus on targeting the wider damage to society associated with political violence, strengthening the family's protective function, and increasing engagement of the folk and professional health care sector.
	Interventions that are introduced from outside the cultural setting undergo a process of adaptation that includes changes in language, therapist–client relationships, use of metaphors, and therapeutic concepts.	
Building on existing resources and resilience	Systematic literature reviews on resilience and protective factors among children in areas of armed conflict [24].	There is tentative evidence suggesting the importance of the family, but on other socioecological levels there is only scant research evidence to support the relationship between resilience and psychosocial well-being among children in areas of armed conflict.
	To strengthen the community resilience component of the system of care, parent–teacher associations have been introduced to mobilize key stakeholders.	

### 1. Multi-Layered Support

Following the overarching aim to develop a care system, an initial focus on individual components of the package gradually shifted to a focus on measuring the impact and feasibility of the overall package. Feasibility (e.g., client satisfaction and cultural sensitivity) and access to care were assessed through practice-driven evaluations. Results allowed for changes to be made in the delivery framework. For example, the level of stress among facilitators and counselors was found to be too high across the settings, which required a change in assigned roles and increased supervision [16]. Furthermore, expenses related to the development and implementation of services were closely monitored to allow cost analyses.

A key challenge of this care system was ensuring that the different interventions were appropriately matched with the target population, and ensuring the successful flow of clients between the components of the system. To facilitate this process of intervention allocation, an instrument was developed to identify children with elevated psychosocial distress. The Child Psychosocial Distress Screener is a multidimensional tool that combines assessment of children's problems (e.g., distress and school absenteeism) and their protective factors (e.g., coping and social support). To ensure context sensitivity across different settings, we developed a core template of seven items, with content of adjoining probes specified in each setting through qualitative enquiry. We first assessed construct and concurrent validity and other psychometric properties within one setting (Burundi), and then assessed construct validity across the different settings using multi-sample confirmatory factor analyses [17],[22]. The assessment confirmed the reliability and validity of the instrument across settings and indicated an optimal cutoff point for detecting children with an indication for psychosocial care. Consequently, the instrument was integrated into the practice of the care package. As a result of screening outcomes, 59% of children were offered the opportunity to participate in recreational peer group activities, and 41% were offered the opportunity to participate in the more therapeutic group intervention (CBI). From the latter group, 7% were referred for counseling or to other mental health specialists (1%). (Percentages are based only on children that entered the screening process, which explains why these numbers differ from those presented in Figure 1.) Through this triage method, we were able to systematically provide access to support or care services to the entire school-going population in the program catchment areas. The down-side of this approach was that children who were not going to school, often a particularly vulnerable group, were not reached. In a later stage of the program this was adjusted by asking key community members to help in the outreach to this group of children.

### 2. Effectiveness of Care

Given the limited evidence base for interventions for children in LMICs, an initial priority was to establish the efficacy of the utilized interventions. Cluster randomized trials in Burundi, Indonesia, Sri Lanka, and Nepal evaluated the efficacy of CBI. Evaluated treatment outcomes included changes in symptoms (e.g., post-traumatic stress disorder, depression, anxiety, and aggression), functional impairment, and positive attitudes and behaviors (hope, prosocial behavior, and coping). Whilst CBI showed efficacy in Indonesia, Sri Lanka, and Nepal (with differential effects across populations and outcome measures) [18]–[20], preliminary results in Burundi did not indicate significant lasting change. As a result of the latter findings, we decided to refocus our intervention efforts in Burundi to target children within their family context. To develop the family-based intervention, we used a new strategy that combined global and local knowledge into a context-sensitive modular intervention. The strategy comprised a qualitative phase to determine intervention objectives, a global expert panel to prioritize intervention modalities, a systematic literature review and distillation of evidence-based treatments, and stakeholder meetings to explore sociocultural acceptability of the intervention [23]. The intervention that we developed through this approach is now being implemented and tested in Burundi and South Sudan.

Besides establishing efficacy, we needed more knowledge on how intervention effects may have been sorted, given the differential effects across settings. For the counseling intervention we explored treatment processes through single case studies. In these studies we aimed to systematically associate client changes (measured with quantitative indicators) to treatment processes (qualitative data), in order to identify treatment processes underlying effective counseling. We learned that counselor performance was highly stable (i.e., there were counselors with consistently positive results and counselors with consistently negative results), which reemphasized the importance of our clinical supervision and evaluation mechanisms, for example, for early identification of counselors performing poorly. Furthermore, results showed that the combination of universal treatment variables (therapeutic alliance and trust, and a non-moralistic and non-normative counselor) with specific treatment components (active problem-solving, narrative exposure, and cognitive restructuring) was associated with positive client trajectories [21]. These findings resulted in changes in the training curricula and led to the exploration of a components-based intervention approach.

### 3. Context Sensitivity

While we used a generic framework across settings, the program was receptive to differences in sociocultural context and care systems. To accommodate these differences, we started the program with qualitative research into how conflict affected children in the different settings, which helped us understand the context and specific needs of the children in the areas where the program was planned [11]. This study established the importance of aspects of community resilience in the different settings. For example, in Burundi, where creating associations is commonly seen as a viable strategy to increase community mobilization, “child-to-child self-help groups” were established as part of the care package.

From practice we learned the importance of adapting psychosocial treatments in new settings. For example, while we had made adaptations in the interventions (including changes in language, therapist–client relationships, use of games and metaphors, and therapeutic concepts), in some settings we were faced with resistance from the community to the group-based intervention (e.g., in Sudan, the group intervention that included hand gestures to show rain and sunshine was perceived as a new religion).

### 4. Utilization of Existing Resources

The systematic literature review we undertook demonstrated the lack of available evidence for primary-prevention-level mental health interventions for children in complex emergencies [24]. Indeed, one of the most challenging aspects of building a care system was to integrate the package within existing community structures and to make use of existing resources. In the program, peer recreational and support groups were included, as explained above. However, more integration is required for a locally relevant and sustainable primary prevention approach. As a result of this, in light of the results of the literature review, we initiated a new strategy within the overall framework, namely, to work with parent–teacher associations in order to promote the psychosocial well-being of children within the school context. Overall, we feel that a shift in emphasis may be required from epidemiology to understanding health systems, when conducting research in conflict-affected settings.

## Looking to the Future

Findings from the different studies presented above have reinforced the notion that a “system of care” approach, however rudimentary, is a necessity not a luxury [25]. We posit that to establish psychosocial and mental health care for children, a care package should be tailored to the context and target population; this can be accomplished by assembling a set of practice components [23],[26]. This approach does not focus narrowly on either a community-based psychosocial intervention (e.g., recreational activities for distressed children) or more specialized psychotherapeutic care (e.g., cognitive behavioral therapy for symptoms of post-traumatic stress disorder); rather, it prioritizes the facilitated transfer of clients between components along a continuum of care, thereby aiming to broadly cover a population at large [2]. Once services are available, non-stigmatizing detection of children in need of care is an integral strategy to improve access. In our case, a two-stage case-identification strategy of school-based detection followed by service-provider-based assessments proved feasible. The accuracy of such a stepped procedure should be evaluated in the future, as it is important in optimizing the match between access to, and provision of, mental health care. To improve upon present limitations to the program, future implementation and research should further include the following: increased emphasis on primary prevention interventions that target resilience at the bottom of the public health pyramid (e.g., integration with other humanitarian initiatives or poverty reduction programs) [8], filling a gap in family approaches [7], increased attention for severe child and adolescent mental disorders (e.g., developmental disorders) through treatment procedures described by the World Health Organization's Mental Health Gap Action Programme [10], and balanced attention to conflict-specific stressors and the burden of daily stressors [27]. Furthermore, future work needs to focus on developing and evaluating strategies to integrate the care package into existing governmental structures to ensure sustainability.

While we promote the current emphasis on accountability and the need to demonstrate the effect of interventions in humanitarian settings, we advocate a broader research agenda that also focuses on care/health system variables, as well as implementation and intervention mechanisms [28].

In the program described here, embedding research in a service delivery framework allowed for generating, and responding to, important implementation issues such as task-shifting, costs of care, burden for caregivers, and how to make interventions culturally compatible.

## Abbreviations

CBI: Classroom-Based Intervention
LMICs: low- and middle-income countries
